# The aryl hydrocarbon receptor ligand omeprazole inhibits breast cancer cell invasion and metastasis

**DOI:** 10.1186/1471-2407-14-498

**Published:** 2014-07-09

**Authors:** Un-Ho Jin, Syng-Ook Lee, Catherine Pfent, Stephen Safe

**Affiliations:** 1 Institute of Biosciences and Technology, Texas A&M Health Sciences Center, 2121 W. Holcombe Blvd., Houston, TX 77030, USA; 2 Department of Veterinary Pathobiology, Texas A&M University, 4466 TAMU, College Station, TX 77843, USA; 3 Department of Veterinary Physiology and Pharmacology, Texas A&M University, 4466 TAMU, College Station, TX 77843, USA

**Keywords:** Omeprazole, Ah receptor, Metastasis, Inhibition, CXCR4

## Abstract

**Background:**

Patients with ER-negative breast tumors are among the most difficult to treat and exhibit low survival rates due, in part, to metastasis from the breast to various distal sites. Aryl hydrocarbon receptor (AHR) ligands show promise as antimetastatic drugs for estrogen receptor (ER)-negative breast cancer.

**Methods:**

Triple negative MDA-MB-231 breast cancer cells were treated with eight AHR-active pharmaceuticals including 4-hydroxtamoxifen, flutamide leflunomide, mexiletine, nimodipine, omeprazole, sulindac and tranilast, and the effects of these compounds on cell proliferation (MTT assay) and cell migration (Boyden chamber assay) were examined. The role of the AHR in mediating inhibition of MDA-MB-231 cell invasion was investigated by RNA interference (RNAi) and knockdown of AHR or cotreatment with AHR agonists. Lung metastasis of MDA-MB-231 cells was evaluated in mice administered cells by tail vein injection and prometastatic gene expression was examined by immunohistochemistry.

**Results:**

We showed that only the proton pump inhibitor omeprazole decreased MDA-MB-231 breast cancer cell invasion *in vitro*. Omeprazole also significantly decreased MDA-MB-231 cancer cell metastasis to the lung in a mouse model (tail vein injection), and *in vitro* studies showed that omeprazole decreased expression of at least two prometastatic genes, namely matrix metalloproteinase-9 (*MMP-9*) and C-X-C chemokine receptor 4 (*CXCR4*). Results of RNA interference studies confirmed that omeprazole-mediated downregulation of *CXCR4* (but not *MMP-9*) was AHR-dependent. Chromatin immunoprecipitation assays demonstrated that omeprazole recruited the AHR to regions in the CXCR4 promoter that contain dioxin response elements (DREs) and this was accompanied by the loss of pol II on the promoter and decreased expression of *CXCR4*.

**Conclusions:**

AHR-active pharmaceuticals such as omeprazole that decrease breast cancer cell invasion and metastasis may have important clinical applications for late stage breast cancer chemotherapy.

## Background

The aryl hydrocarbon receptor (AHR) is a ligand-activated transcription factor that was first discovered as an intracellular protein that bound with high affinity to the environmental toxicant 2,3,7,8-tetrachlorodibenzo-*p*-dioxin (TCDD) [1]. Subsequent studies showed that AHR-mediated transcription was dependent on formation of a nuclear heterodimer composed of the AHR and AHR nuclear translocator (ARNT) proteins [2] that bind AHR responsive elements (AhREs) on target gene promoters [3]. Initial studies demonstrated that TCDD and structurally-related halogenated aromatic compounds induced a well-defined subset of genes and toxic responses [4]. However, it is now apparent that this receptor plays a critical endogenous role in cellular homeostasis and multiple diseases and binds not only toxicants but also endogenous biochemicals, dietary flavonoids and several phytochemicals associated with health benefits, other synthetic/industrial chemicals, and many pharmaceuticals [5-7]. The important role of the AHR and effects of AHR agonists or antagonists have been documented for various inflammatory conditions, stem cell stability and expansion, autoimmune diseases, and several different cancers and clearly demonstrate that this receptor is an important drug target [8-15].

Research in this laboratory initially focused on the molecular mechanisms of inhibitory AHR-estrogen receptor (ER) crosstalk and development of selective AHR modulators (SAhRMs) for treatment of ER-positive breast cancer [16,17]. 6-Methyl-1,3,8-trichlordibenzofuran (6-MCDF) was initially developed as a relatively non-toxic AHR antagonist that inhibited TCDD-induced toxicity in rodent models [18-22]. However, this compound also exhibited AHR agonist activity and activated inhibitory AHR-ERα crosstalk in breast cancer cells and decreased mammary tumor growth *in vivo*[17,23,24]. Subsequent studies showed that MCDF also blocked growth of ER-negative breast cancer cells [25] and inhibited metastasis of triple negative MDA-MB-231 breast cancer cells to the lung by inducing the antimetastatic microRNA-335 (miR-335) [26]. Recent studies showed that eight AHR-active pharmaceuticals including 4-hydroxtamoxifen, flutamide leflunomide, mexiletine, nimodipine, omeprazole, sulindac and tranilast exhibited structure- and cell context-dependent AHR agonist/antagonist activities in BT474 and MDA-MB-468 cells and several of these compounds also inhibited MDA-MB-468 cell migration [27]. These results are typically observed for selective AhR modulators (SAhRMs) that exhibit tissue- and response-specific AhR agonist or antagonist activity due to differential expression of cofactors, different receptor/ligand conformations and epigenetic effects [16]. Selective receptor modulators are also commonly observed for nuclear receptors such as the estrogen receptor (ER) and selective ER modulators have been extensively characterized for treatment of ER-positive breast cancer [28].

In this study, we initially used the same set of AHR-active pharmaceuticals in triple-negative MDA-MB-231 cells with a primary objective of identifying a known pharmaceutical that may be effective for inhibiting breast cancer metastasis. Among the eight compounds, only omeprazole inhibited MDA-MB-231 breast cancer cell invasion and this response could be reversed, in part, by AHR antagonists or by knockdown of the AHR by RNA interference (RNAi). Omeprazole also inhibited lung metastasis of MDA-MB-231 cells (tail vein injection) in a mouse model and the antimetastatic pathway was linked to decreased expression of *MMP-9* and AHR-dependent suppression of the pro-metastatic gene *CXCR4*. Decreased invasion and *CXCR4* expression was also observed in MCF-7 and SKBR3 breast cancer cell lines treated with omeprazole. Thus, omeprazole may have potential clinical applications for inhibition of breast cancer metastasis due, in part, to its AHR agonist activity.

## Methods

### Cell lines, antibodies, and reagents and MTT assay

MDA-MB-231, MCF-7, SKBR3 and MDA-MB-468 human breast cancer cell lines were obtained from the American Type Culture Collection (Manassas, VA). Cells were maintained in Dulbecco’s modified Eagle’s medium (DMEM) nutrient mixture supplemented with 0.22% sodium bicarbonate, 0.011% sodium pyruvate, 10% fetal bovine serum (FBS), and 10 ml/L 100× antibiotic/antimycotic solution (Sigma-Aldrich, St. Louis, MO). Cells were maintained at 37°C in the presence of 5% CO_2_, and the solvent (dimethyl sulfoxide, DMSO) used in the experiments was ≤0.2%. CYP1A1, AHR, PCNA, and β-actin antibodies were purchased from Santa Cruz Biotechnology (Santa Cruz, CA), and CXCR4 and RNA polymerase II antibody were purchased from GeneTex (Irvine, CA). All compounds used in this study and reagents for cell staining and MTT assay were purchased from Sigma-Aldrich. Cells (5 × 10^3^ per well) were plated in 96-well plates and allowed to attach for 16 hr, and the effects of various AHR-active compounds on cell proliferation were determined in an MTT assay as previously described [27].

### Chromatin immunoprecipitation (ChIP) assay

The ChIP assay was performed using ChIP-IT Express Magnetic Chromatin Immunoprecipitation kit (Active Motif, Carlsbad, CA) according to the manufacturer’s protocol. MDA-MB-231 cells (5 × 10^6^ cells) were treated with TCDD or omeprazole for 2 hr, and the ChIP assay was carried out as previously described [27]. The CXCR4-123 primers were 5′- ATC CCT GGC ATT TCA TCT CTC C-3′ (sense) and 5′- ACA ACA CCG TGT GGG TAT TAC C-3′ (antisense) and the CXCR4-4 primers were 5′- ACT CAC TAC CGA CCA CCC GC-3′ (sense) and 5′- CGT CAC TTT GCT ACC TGC TGC C-3′ (antisense), and then respectively amplified a 171-bp and 232-bp region of human CXCR4 promoter which contained the AHR binding sequences. The cytochrome P4501A1 (CYP1A1) primers were 5′-TCA GGG CTG GGG TCG CAG CGC TTC T-3′ (sense), and 5′-GCT ACA GCC TAC CAG GAC TCG GCA G-3′ (antisense), and then amplified a 122-bp region of human CYP1A1 promoter which contained the AHR binding sequences [27]. PCR products were resolved on a 2% agarose gel in the presence of ETBR.

### Quantitative real-time PCR

cDNA was prepared from the total RNA of cells using High Capacity RNA-to-cDNA Kit (Applied Biosystems, Foster City, CA) as previously described [27]. Values for each gene were normalized to expression levels of TATA-binding protein. The sequences of the primers used for real-time PCR were as follows: CYP1A1 sense 5′- GAC CAC AAC CAC CAA GAA C-3′, antisense 5′- AGC GAA GAA TAG GGA TGA AG-3′; cytochrome P4501B1 (CYP1B1) sense 5′- ACC TGA TCC AAT TCT GCC TG-3′, antisense 5′- TAT CAC TGA CAT CTT CGG CG-3′; CXCR4 sense 5′- TTT TCT TCA CGG AAA CAG GG-3′, antisense 5′- GTT ACC ATG GAG GGG ATC AG-3′; MMP-9 sense 5′- TTG GTC CAC CTG GTT CAA CT-3′, antisense 5′- ACG ACG TCT TCC AGT ACC GA-3′; and TBP sense 5′-TGC ACA GGA GCC AAG AGT GAA-3′, antisense 5′-CAC ATC ACA GCT CCC CAC CA-3′.

### Western blot analysis

Cells (3 × 10^5^) were plated in 6-well plates in DMEM media containing 2.5% FBS for 16 hr and then treated with different concentrations of the compounds, and whole cell lysates were analyzed by western blots essentially as described [27].

### Scratch and invasion assay

After cells were more than 80% confluent in 6-well plates, the scratch was made using a sterile pipette and then treated with vehicle (DMSO) or compounds. Cell migration into the scratch was determined after 18 hr (7-8 determinations/treatment). For invasion assay of MDA-MB-231 cells, the BD-Matrigel Invasion Chamber (24-transwell with 8 μm pore size polycarbonate membrane) was used in a process of modified Boyden chamber assay essentially as described [27].

### Transfection of siRNAs and luciferase assays

Cells (2 × 10^5^ cells/well) were plated in 6-well plates in DMEM media supplemented with 10% FBS. After 16 hr, the cells were transfected with 100 nM of each siRNA duplex for 6 hr using Lipofectamine 2000 reagent (Invitrogen) following the manufacturer’s protocol essentially as described [27]. In the AhR knockdown experiments, cells were transfected with AhR siRNA or a non-specific (control) oligonucleotide [25-27]. The CXCR4 (NM_003467) promoter clone (CXCR4 promoter-Gaussia luciferase reporter construct containing secreted alkaline phosphatase) and Secrete-Pair Gaussia Luciferase Assay Kit were purchased from Genecopoeia (Rockville, MD). Cells (4 × 10^4^ cells/well) were plated in 12-well plates in DMEM media supplemented with 10% FBS and transfection experiments were carried out as described [27]. A multifunctional microplate reader (FLUOstar OPTIMA) was used to quantitate luciferase and phosphatase activities, and the luciferase activities were normalized to alkaline phosphatase activity.

### Tail vein injection for metastasis in athymic mice and immunohistochemistry

Female athymic nude mice (*Foxn1*^*nu*^, ages 6-8 weeks) were purchased from Harlan Laboratories. Animal work was approved by the Institutional Animal Care and Use Committee (IACUC) at Texas A&M University. MDA-MB-231 cells (1 × 10^6^ cells) in PBS were injected through the tail vein of nude mice to create pulmonary metastasis, and mice were randomly divided into 2 groups of 6 animals each. Either corn oil (control) or omeprazole (100 mg/kg/day) in corn oil was orally administered to each group for 4 weeks, respectively. The lung tissues were fixed in 10% neutral buffered formalin and further examined by routine (H&E) and immonohistochemical staining. Paraffin-embedded lung tissue sections (5-μm thick) were analyzed for CXCR4 and proliferating cell nuclear antigen (PCNA) as previously described [27].

### Statistics

All of the experiments were repeated a minimum of three times. The data are expressed as the means ± SE. Statistical significance was analyzed using either Student’s t-test or analysis of variance (ANOVA) with Scheffe’s test. The results are expressed as means with error bars representing 95% confidence intervals for three experiments for each group unless otherwise indicated, and a *P* value of less than 0.05 was considered statistically significant.

## Results

### Omeprazole inhibits MDA-MB-231 cell invasion

**Figure 1:**
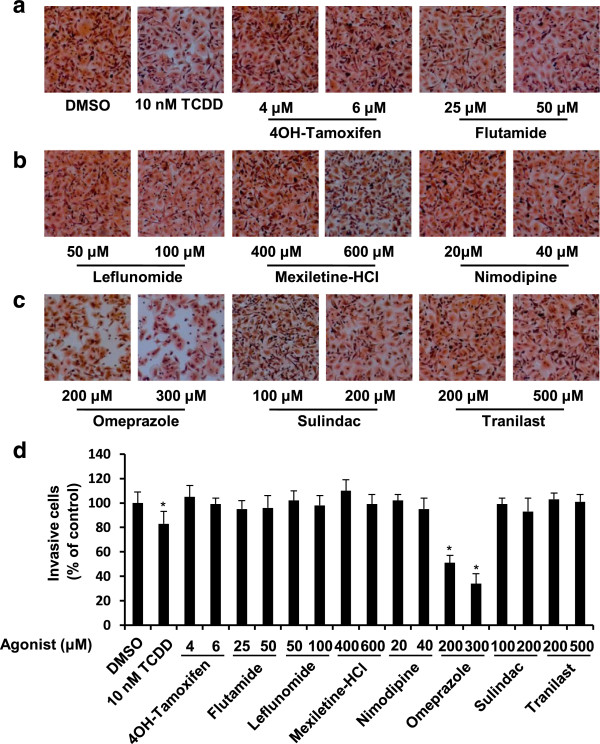
**Omeprazole inhibits MDA-MB-231 cell invasion. (a)-(c)** MDA-MB-231 cells were treated with omeprazole, other AHR-active pharmaceuticals and TCDD, and MDA-MB-231 cancer cell invasion was determined using a Boyden chamber assay as outlined in the Methods. **(d)** Quantitation of drug-induced inhibition of invasion. Experiments outlined in **(a)-(c)** were determined in triplicate and results are expressed as means ± SE. Significant (p < 0.05) inhibition of invasion is indicated.

Table 1 and Additional file 1: Figure S1, Additional file 2: Figure S2 and Additional file 3: Figure S3 show that the eight AHR-active pharmaceuticals differentially activated CYP1A1 and CYP1B1 mRNA levels in MDA-MB-231 cells, and only 4-hydroxytamoxifen induced > 50% of the maximal response for both genes compared to 10 nM TCDD (100% response). Induction of CYP1A1 and CYP1B1 are prototypical markers of AH-responsiveness of cells to TCDD and other AHR agonists. Induction of CYP1A1 protein by these compounds was variable and 4-hydroxytamoxifen did not induce this response. TCDD typically induces proteasome-dependent degradation of the AhR and this was observed in MDA-MB-231 cells (Additional file 3: Figure S3). The effects of the AhR pharmaceuticals on AhR levels were highly variable, and notable decreases were observed for leflunomide, nimodipine, sulindac and 4-hydroxytamoxifen and this did not correlate with their effects on other measures of Ah-responsiveness. The variable response patterns observed for these compounds in MDA-MB-231 cells paralleled their structure- and cell context-dependent variability as AHR agonists and antagonists as previously observed in MDA-MB-468 and BT474 cells [27]. However, treatment of MDA-MB-231 cells with the AHR-active pharmaceuticals using concentrations of each compound that were not cytotoxic (≤20% growth inhibitory effect) (Additional file 4: Figure S4) showed that among these compounds, only 200 and 300 μM omeprazole inhibited MDA-MB-231 cell migration in a Boyden chamber assay (Figure 1).

**Table 1:** AHR-active pharmaceuticals as AHR agonists in MDA-MB-231 breast cancer cells

**AHR agonist**	**mRNA**		**Protein**	
	**CYP1A1**	**CYP1B1**	**CYP1A1**	**AHR**
40-Hydroxytamoxifen	>50	>50	ni	decreased
Sulindac	<50	>50	ni	decreased
Flutamide	<50	>50	ni	unchanged
Tranilast	<50	<50	ind	decreased
Leflunomide	<50	>50	ind	decreased
Nimodipine	<50	>50	ind	decreased
Mexiletine	<50	>50	ind	decreased
Omeprazole	<50	<50	ind	decreased

>50 + <50 are the % of TCDD-induced, response (100%).

ni = no induction; md = induction.

### Inhibition of breast cancer cell invasion and metastasis: role of the AHR

**Figure 2:**
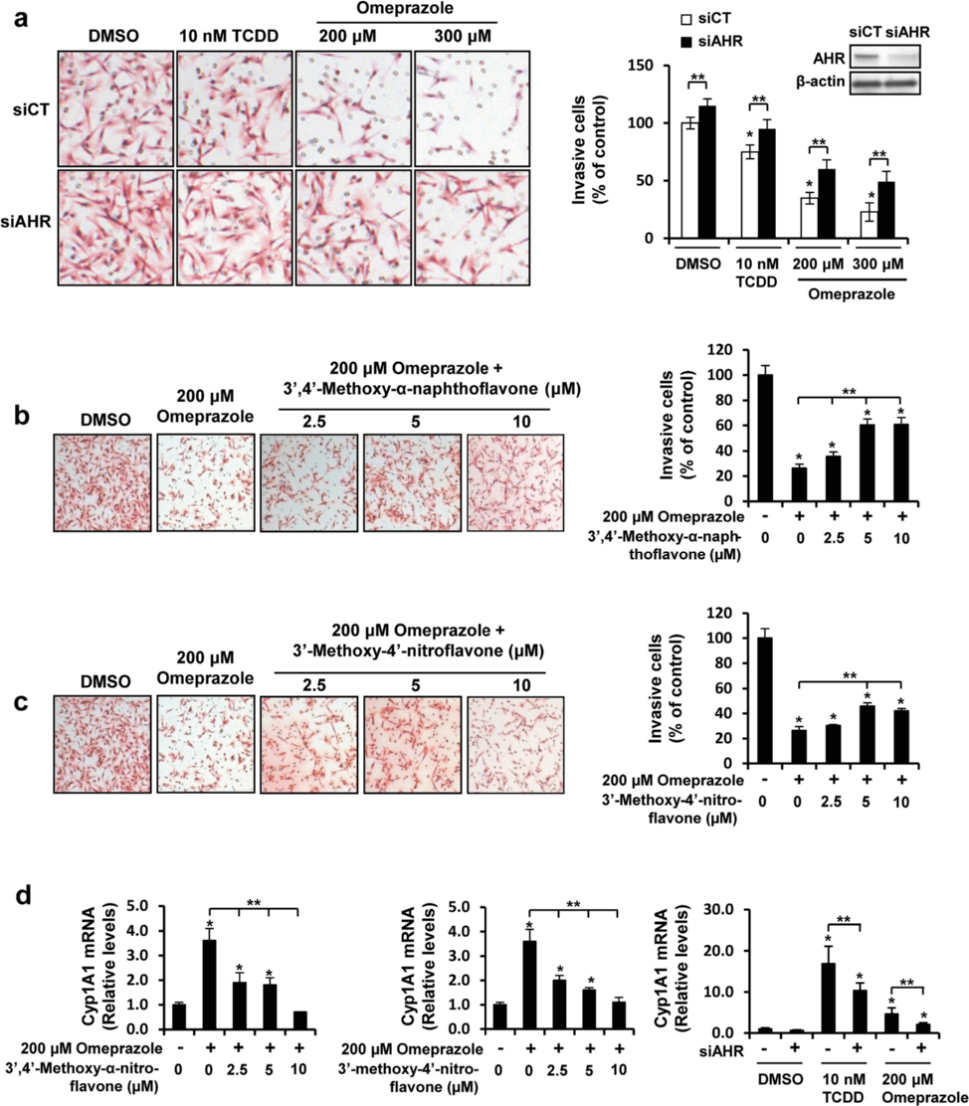
**Role of the AHR in mediating inhibition of MDA-MB-231 cell invasion by omeprazole. (a)** AHR silencing. MDA-MB-231 cells were transfected with siCtl (control) and siAHR (targeting AHR) oligonucleotides treated with DMSO, TCDD or omeprazole, and effects on cell invasion were determined in a Boyden Chamber assay as outlined in the Methods. Significant (p < 0.05) inhibition of invasion (*) and reversal of these effects by siAHR (**) are indicated. Similar results were observed with another siAHR oligonucleotide (data not shown). AHR antagonists 3′,4′-dimethoxy-α-naphthoflavone **(b)** and 3′-methoxy-4′-nitroflavone **(c)** block omeprazole-induced effects on invasion. Cells were treated with DMSO, omeprazole and the AHR antagonists alone or in combination, and cell invasion was determined in a Boyden chamber assay as outlined in the Methods. Significant (p < 0.05) inhibition of invasion (*) and rescue by the AHR antagonists (**) is indicated. **(d)** AHR antagonist and silencing inhibits induction of CYP1A1 mRNA by omeprazole. Cells were treated with DMSO, omeprazole and the antagonists alone or in combination (right and middle panel) or transfected with siCtl or siAHR and treated with DMSO, TCDD or omeprazole, and CYP1A1 mRNA levels were determined by real time PCR as outlined in the Methods. Significant (p < 0.05) induction of CYP1A1 (*) and reversal of this effect (**) are indicated. Results (a - d) are expressed as means ± SE for at least 3 replicate experiments for each treatment group.

The role of the AHR in mediating inhibition of MDA-MB-231 cell invasion was investigated by RNA interference (RNAi) and cotreatment with AHR agonists. Figure 2A shows that TCDD- and omeprazole-mediated inhibition of invasion was significantly reversed in cells transfected with a small inhibitory RNA against the AHR (siAHR) compared to a control oligonucleotide (siCT). Knockdown of the AHR alone also significantly increased MDA-MB-231 cell invasion, suggesting that the receptor alone inhibited invasion. Similar results were observed in MDA-MB-231 cells cotreated with omeprazole and the AHR antagonists 3′,4′-methoxy-α-naphthoflavone (Figure 2B) [29] and 3′-methoxy-4′-nitroflavone (Figure 2C) [30], further confirming a role for the AHR in mediating the inhibitory effects of omeprazole on MDA-MB-231 breast cancer cell invasion. As a positive control, we also showed that AHR knockdown and the AHR antagonists also inhibited induction of CYP1A1 by TCDD and omeprazole (Figure 2D).

**Figure 3:**
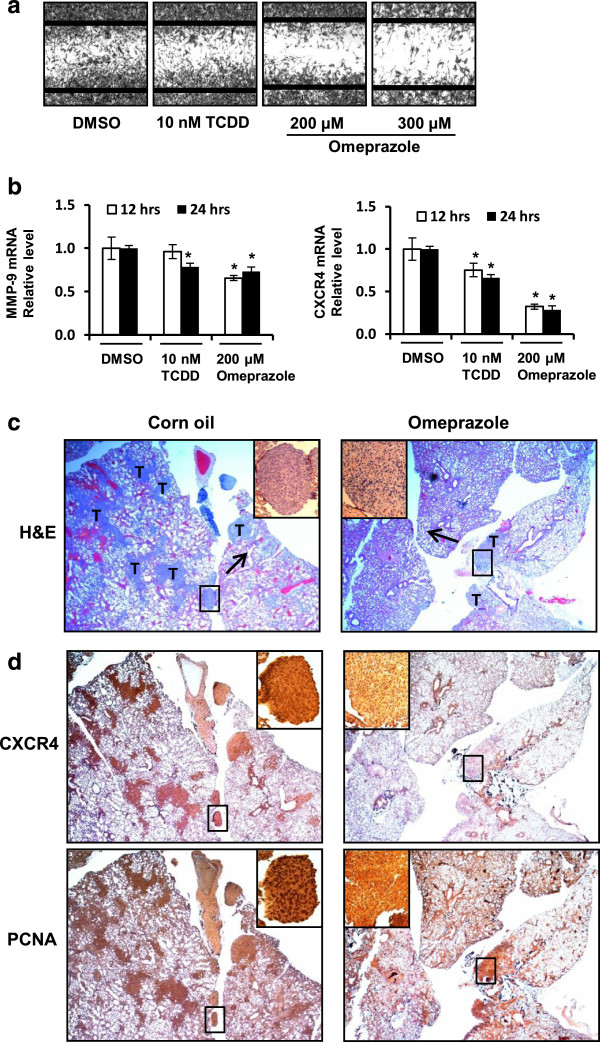
**Omeprazole inhibits migration and invasion *in vitro *and metastasis *in vivo*. (a)** Migration. MDA-MB-231 cells were treated with DMSO, TCDD or omeprazole, and cell migration was determined using a scratch assay as outlined in the Methods. **(b)** Decreased MMP-9 and CXCR4 expression. MDA-MB-231 cells were treated with DMSO, TCDD or omeprazole, and MMP-9 and CXCR4 mRNA levels were determined by real time PCR as outlined in the Methods. Results are means ± SE for at least 3 replicate determinations and significant (p < 0.05) inhibition is indicated. H&E staining **(c)** and immunostaining **(d)** of lung tissue from *in vivo* studies. Lung tissue sections from animals treated with corn oil (control) or omeprazole (100 mg/kg/d) were obtained for H&E and immunostaining as outlined in the Methods. Image magnification 40×; inset magnification 100 × .

TCDD (10 nM) and omeprazole (200 and 300 μM) significantly decreased MDA-MB-231 cell migration in a scratch assay (Figure 3A) and these results paralleled the inhibition MDA-MB-231 cell invasion after treatment with omeprazole. We also screened for expression of several genes associated with cancer cell invasion and metastasis and observed that omeprazole and TCDD decreased expression of *MMP-9* and *CXCR4* (Figures 3B), and the latter pro-metastatic gene has previous been shown to be repressed by TCDD and other AHR ligands in breast cancer cells [31-34]. The antimetastatic activity of omeprazole was also investigated in athymic nude mice injected (tail vein) with MDA-MB-231 cells and treated with 100 mg/kg/d omeprazole for 28 days. Treatment-related weight loss was not observed, and H&E staining was used to evaluate and quantify the total number of tumor cells relative to the number of pneumocytes. In vehicle control mice, 9,785 tumor cells were counted in fifty frames with 46,653 pneumocytes (21.0%), whereas in omeprazole-treated mice, 3,552 tumor cells were counted with 43,086 pneumocytes (8.2%). These results demonstrate that omeprazole significantly inhibited lung metastasis of MDA-MB-231 cells by tail vein injection and this is illustrated in Figure 3C. Lung tumors were also immunostained for CXCR4 and PCNA (Figure 3D) and the decreased expression of both markers in the metastasized tumors in omeprazole-treated mice, suggesting that omeprazole also directly affected these tumors after metastasis to the lung.

**Figure 4:**
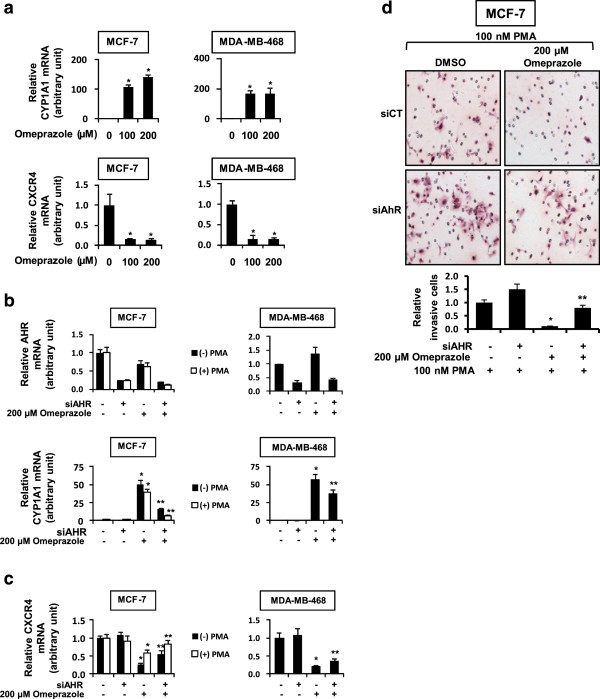
**Role of omeprazole and the AHR in downregulation of *CXCR4 *and inhibition of invasion in MCF-7 and MDA-MB-468 cells. (a)** CYP1A1 and CXCR4 mRNA. Cells were treated with 100 or 200 μM omeprazole for 24 hr, and CXCR4 and CYP1A1 mRNA levels were determined as outlined in the Methods. **(b)** and **(c)** Role of the AHR. Cells were transfected with siAHR and treated with 200 μM omeprazole, and expression of AHR and CYP1A1 **(b)** and CXCR4 **(c)** mRNA levels were determined as outlined in the Methods. MCF-7 cells were also cotreated with 100 nM PMA. **(d)** Effects of omeprazole on invasion of MCF-7 cells. Cells were transfected with siCtl (non-specific) or siAHR and treated with DMSO or 200 μM omeprazole plus PMA (MCF-7 cells), and cell invasion was determined in a Boyden chamber assay as outlined in the Methods. Results **(a-d)** are means ± SE for at least 3 replicate determinations for each data point. Significantly (p < 0.05) increased CYP1A1 or decreased CXCR4 (*) and reversal of these effects by siAHR (**) are indicated. AHR mRNA was significantly (p < 0.05) decreased by siAHR in all 3 cell lines.

Omeprazole also induced CYP1A1 and decreased CXCR4 mRNA levels in MCF-7 and MDA-MB-468 cells (Figure 4A). Knockdown of the AHR in these cell lines decreased AHR mRNA levels and significantly reversed omeprazole-mediated induction of CYP1A1 (Figure 4B) and inhibition of CXCR4 (Figure 4C) mRNA levels in both cell lines. Omeprazole inhibits MDA-MB-468 cell migration [27]; however, this cell line did not exhibit invasion. PMA induced invasion of MCF-7 cells and this response was inhibited by omeprazole and partially reversed in cells transfected with siAHR (Figure 4C). Thus, the effects of omeprazole in the two AH-responsive MCF-7 and MDA-MB-468 cell lines were similar to that observed in MDA-MB-231 cells.

### Repression of CXCR4 expression by omeprazole is AHR-dependent

**Figure 5:**
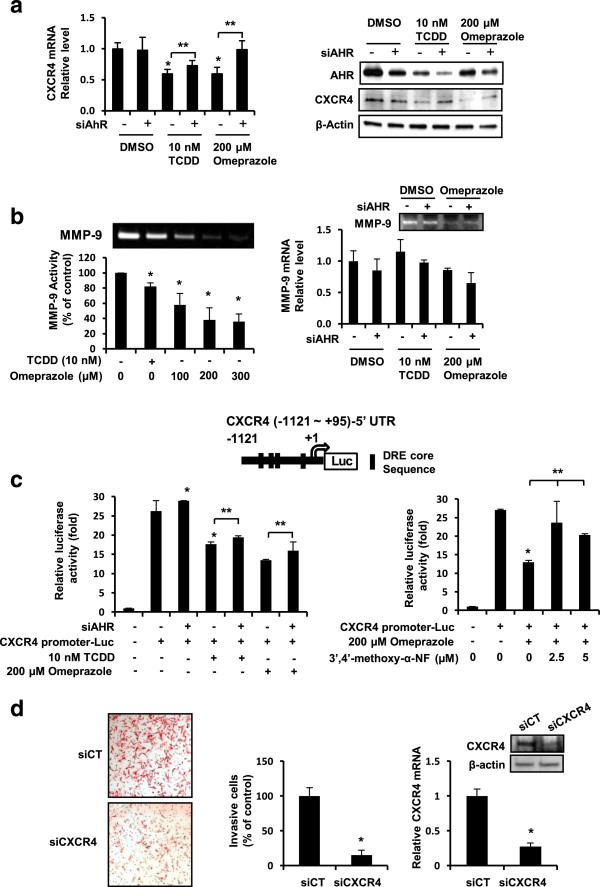
**Omeprazole and TCDD decrease CXCR4 and MMP-9. (a)** CXCR4 mRNA and protein. MDA-MB-231 cells were treated with DMSO, omeprazole or TCDD and transfected with siCtl (control) or si AHR, and mRNA and protein levels were determined as described in the Methods. **(b)** Effects on MMP-9. Cells were treated with DMSO, TCDD or omeprazole, and MMP-9 activity (zymography) and MMP-9 mRNA levels (in cells transfected with siCtl or siAHR) were determined as outlined in the Methods. **(c)** CXCR4 promoter activity. MDA-MB-231 cells were transfected with CXCR4-(-1121 to +95)luc and either cotransfected with siCtl and siAHR or treated with 3′,4′-dimethoxy-α-naphthoflavone and luciferase activity determined as outlined in the Methods. **(d)** Silencing of CXCR4. Cells were transfected with siCtl or siCXCR4 and effects on cancer cell invasion and CXCR4 expression were determined as outlined in the Methods. Results **(a-d)** are means ± SE for at least 3 experiments and significant (p < 0.05) decrease (*) or rescue (**) is indicated.

Results in Figure 5A show that omeprazole and TCDD decreased expression of CXCR4 mRNA levels in MDA-MB-231 cells and this was partially reversed in cells transfected with siAHR, and similar results were observed for CXCR4 protein. Omeprazole and TCDD decreased MMP-9 activity (by zymography and mRNA); however, AHR silencing did not attenuate downregulation of MMP-9 mRNA levels (Figure 5B, right panel), suggesting that downregulation of MMP-9 was AHR-independent. TCDD and omeprazole decreased luciferase activity in MDA-MB-231 cells transfected with the CXCR4-luc construct which contains the -1121 to +95 region of the CXCR4 promoter (Figure 5C). TCDD- and omeprazole-induced downregulation of luciferase activity was significantly reversed in cells cotransfected with siAHR or cotreated with the AHR antagonist 3′4′-dimethoxy-α-naphthoflavone (Figure 5C), confirming a role for the AHR in mediating this response. The role of CXCR4 downregulation in mediating the inhibitory effects of omeprazole on MDA-MB-231 cell invasion was supported by results showing that knockdown of CXCR4 by RNAi also decreased invasion of MDA-MB-231 cells (Figure 5D).

**Figure 6:**
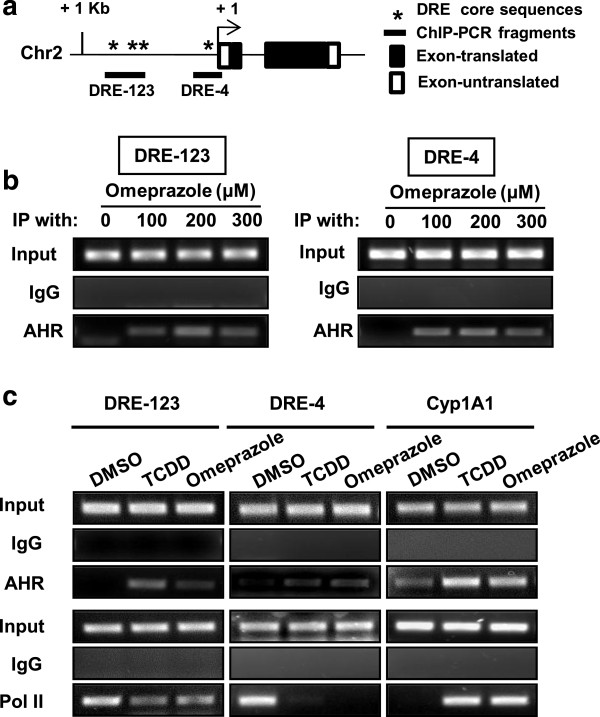
**ChIP assays and AHR-DRE binding. (a)** Schematic outline of DRE-123 and DRE-4 on the CXCR4 promoter. ChIP analysis of **(b)** omeprazole and **(c)** omeprazole and TCDD-induced interactions with the DRE-123, DRE-4 and CYP1A1-DRE *cis*-elements. MDA-MB-231 cells were treated with DMSO, omeprazole and TCDD, and binding to various DRE elements was determined in ChIP assays as described in the Methods.

The proximal Ah-responsive region of the CXCR4 promoter contains 4 putative DREs with a core GCGTG AHR complex binding motif (Figure 6A), and primers for the upstream DRE-123 and downstream DRE-4 region of the promoter were used for determining recruitment or loss of AHR binding in a ChIP assay. Omeprazole (100-300 μM) clearly induced AHR binding to DRE-123 and DRE-4 and, in the untreated cells, binding of this receptor was not observed at either DRE site (Figure 6B). The comparative effects of 10 nM TCDD and 200 μM omeprazole on inducing AHR binding to DRE-123 and DRE-4 in the CXCR4 promoter and the CYP1A1 DRE was also investigated in MDA-MB-231 cells, and both ligands induced AHR binding at all 3 *cis*-DRE motifs (Figure 6C). In contrast, omeprazole and TCDD decreased binding of pol II to the CXCR4 promoter but increased pol II binding to the CYP1A1 gene promoter and this corresponded to the ligand-mediated suppression and induction of these genes, respectively. Recruitment of the corepressor SMRT to the CXCR4 promoter was not observed (data not shown), and the role of other nuclear cofactors in mediating AHR-dependent downregulation of *CXCR4* by TCDD and omeprazole is currently being investigated.

## Discussion

The treatment and prognosis for breast cancer patients depends on multiple tumor characteristics including the size, stage, extent of tumor delocalization, and expression of various protein and mRNA prognostic factors [35]. Patients that express ERα can be successfully treated with antiestrogens and aromatase inhibitors, and more aggressive tumors that overexpress the epidermal growth factor receptor 2 (*ErbB2*) oncogene can be treated with the ErbB2 neutralizing antibody in combination therapies [36]. Patients with ER-negative tumors are among the most difficult to treat and exhibit low survival rates due, in part, to metastasis from the breast to various distal sites.

Research in this laboratory and others [33,34] have demonstrated that the AHR is a potential drug target for treating ER-negative breast cancer. For example, the SAhRM MCDF acts as an AhR agonist to inhibit growth and/or metastasis of ER-negative breast tumors in animal models, and this was associated with induction of microRNA-335 and the subsequent suppression of the pro-metastatic *SOX4* gene [26]. Several other studies with structurally diverse AHR ligands (agonists) demonstrated AHR-mediated inhibition of cell migration and/or invasion in ER-negative breast cancer cells and these responses were also accompanied by enhanced differentiation and downregulation of pro-metastatic genes such as *CXCR4* and *MMP-9*[31-34,37-39].

A number of pharmaceutical agents approved for multiple uses are also AHR ligands, and some of these compounds including 4-hydroxytamoxifen and tranilast exhibit some anticancer activity in breast cancer cells [37-39]. Our initial studies investigated the AHR agonist activities of eight AHR-active pharmaceuticals including tranilast and 4-hydroxytamoxifen in MDA-MB-468 and BT474 breast cancer cells and observed that their AHR activity was structure-, cell context- and response-specific [27]. This variability in activity is illustrated by results for mexiletine which was an AHR antagonist in MDA-MB-468 cells and inhibited TCDD-induced *CYP1A1* gene expression but was a partial AHR agonist in BT474 cells [27]. Moreover, in ER-negative MDA-MB-468 cells, several of the AHR pharmaceuticals including flutamide, leflunomide, nimodipine, omeprazole, sulindac and tranilast inhibited cell migration [27]. In this study, we primarily focused on the effects of the AHR-active pharmaceuticals in the more aggressive MDA-MB-231 cell line which exhibits high basal rates of migration and also invasion in *in vitro* assays. However, we also observed that omeprazole induced CYP1A1 in MCF-7 and MDA-MB-468 cells (Figure 4A) [27]. The effects of the AHR-active pharmaceuticals on CYP1A1 and CYP1B1 expression in MDA-MB-231 cells were similar (Table 1); however, only omeprazole inhibited MDA-MB-231 cell invasion (Figures 1D and 2A) and we therefore selected this widely used proton pump inhibitor for further evaluation as an AHR-active antimetastatic agent in breast cancer. Previous studies suggest that omeprazole exhibits anti-inflammatory activity [40] and anticancer activity, particularly in combination treatment studies [41-43]. Since this investigation focused primarily on the effects of omeprazole alone, higher concentrations that were not cytotoxic were used in the cell culture experiments. Previous *in vivo* studies used an omeprazole dose of 75 mg/kg for administration of drug combinations and this dose had no effect on tumor growth [41]. A 100 mg/kg (daily) dose was used for investigating the *in vivo* antimetastic activity of omeprazole (Figure 3C,D).

Omeprazole clearly inhibited MDA-MB-231 cell migration and invasion (Figures 2A and 3A) and this response was attenuated after knockdown of the AHR by RNAi or after cotreatment with AHR antagonists (Figure 2A-C). MDA-MB-468 cells did not exhibit invasion; however, omeprazole inhibited PMA-induced invasion of MCF-7 cells and this response was also attenuated after AHR knockdown (Figure 4D). Moreover, these *in vitro* assays were complemented by inhibition of lung metastasis of MDA-MB-231 cells in mice administered the cells by tail vein injection and treated with omeprazole (Figure 3B). In contrast to the effects observed for MCDF [26], omeprazole did not induce miR-335 expression in MDA-MB-231 (data not shown), but significantly decreased expression of the pro-metastatic genes *MMP-9* and *CXCR4* (Figure 5A,B) and similar results were observed in MCF-7 and MDA-MB-468 cells (Figure 4C). Previous studies show that one or both of these genes was decreased in ER-positive cells and tumors treated with structurally diverse AHR ligands [31-34]. We also observed that *CXCR4* (and *PCNA*) expression was also decreased in metastasized tumors (lung) (Figure 3D), suggesting that omeprazole not only decreased metastasis but directly targeted *CXCR4* and *PCNA* in the metastasized tumors. We further investigated the role of the AHR in mediating the induction of *MMP-9* and *CXCR4* by omeprazole in MDA-MB-231 cells and our results show that although AHR silencing may decrease *MMP-9* activity (zymography), the loss of the receptor does not attenuate the effects of omeprazole on *MMP-9* (Figure 4B). In contrast, omeprazole-induced downregulation of *CXCR4* was significantly reversed by AHR silencing or cotreatment with AHR antagonists (Figure 5A,C), suggesting that downregulation of *MMP-9* and *CXCR4* by omeprazole in MDA-MB-231 cells is AHR-independent and -dependent, respectively.

Although the AHR and other nuclear receptors mediate induction and expression of genes, most mechanistic studies have focused on activation of genes and the ligand-dependent recruitment of the AHR and nuclear cofactors to DREs in Ah-responsive gene promoters [44]. Treatment of MDA-MB-231 cells with omeprazole or TCDD resulted in recruitment of the AHR to the CYP1A1 gene promoter and this was accompanied recruitment of pol II (Figure 6C) and induction of *CYP1A1* gene expression (Additional file 1: Figure S1 and Additional file 1: Figure S3). The CXCR4 promoter contains two regions with *cis*-elements consistent with DRE sequences (DRE-123 and DRE-4). Omeprazole decreased luciferase activity in MDA-MB-231 cells transfected with the pGL3-CXCR4 (-1121 to +95) construct which contains these DREs and this response was attenuated by AHR silencing or AHR antagonists (Figure 4C). Omeprazole and TCDD also induced AHR binding to the CXCR4 promoter (Figure 6B,C); however, in contrast to the recruitment of pol II to the CYP1A1 promoter, both TCDD and omeprazole decreased pol II interactions with the CXCR4 promoter (Figure 6C) and this was consistent with omeprazole-mediated repression of *CXCR4* gene expression. We did not observe ligand-dependent recruitment of the corepressor SMRT to the CXCR4 promoter and are currently investigating the role of other nuclear cofactors required for AHR-mediated suppression of *CXCR4* and other genes in cancer cells treated with omeprazole. Thus, the anticancer activity of omeprazole is due, in part, to the AhR but this does not exclude a role for other AhR-independent pathways.

## Conclusions

In summary, results of this study demonstrate that among several AHR-active pharmaceuticals, omeprazole exhibits antimetastatic activity for triple-negative MDA-MB-231 breast cancer cells, and *CXCR4* is one of the key target genes not only for omeprazole but also for other AHR agonists [31-34]. AHR-dependent downregulation of *CXCR4* by omeprazole significantly contributed to the antimetastatic activity of this compound since silencing of *CXCR4* by RNAi in MDA-MB-231 cells also inhibited invasion of these cells in a Boyden chamber assay (Figure 5D). Since *CXCR4* has both functional and prognostic significance for metastasis in breast tumors and cells [45], the antimetastatic activity of omeprazole and other AHR-active pharmaceuticals including other benzimidazole protein pump inhibitors are currently being investigated. The anticancer activity of drugs used for treatment of gastroesophageal reflux disease is not well established [46] and it is possible that chemotherapeutic effects of omeprazole for inhibition of breast cancer metastasis may require higher doses than are typically used for treating acid reflux. However, lower doses of omeprazole may be effective for drug combination therapies [41] and these are currently being investigated.

## Abbreviations

6-MCDF: 6-methyl-1,3,8-trichlorodibenzofuran; AHR: Aryl hydrocarbon receptor; AhREs: Aryl hydrocarbon response elements; ARNT: Aryl hydrocarbon receptor nuclear translocator; ChIP: Chromatin immunoprecipitation; DMEM: Dulbecco’s modified Eagle’s medium; DMSO: Dimethyl sulfoxide; DREs: Dioxin response elements; ER: Estrogen receptor; FBS: Fetal bovine serum; miR-335: MicroRNA-335; RNAi: RNA interference; SAhRMs: Selective aryl hydrocarbon receptor modulators; TCDD: 2,3,7,8-tetrachlorodibenzo-*p*-dioxin.

## Competing interest

The authors declare that have no competing interest.

## Authors’ contributions

UHJ carried out most of the *in vivo* and *in vitro* studies. SOL assisted UHJ in the *in vivo* studies and the immunostaining experiments. CP carried out the pathology studies. SS supervised the project and wrote the paper. All authors read and approved the final manuscript.

## Pre-publication history

The pre-publication history for this paper can be accessed here:

http://www.biomedcentral.com/1471-2407/14/498/prepub

## Supplementary Material

Additional file 1: Figure S1 Induction of CYP1A1 mRNA by AHR-active pharmaceuticals. MDA-MB-231 cells were treated with DMSO, different concentrations of pharmaceuticals and 10 nM TCDD, and CYP1A1 mRNA levels were determined by real time PCR as outlined in the Methods. Results are expressed as means ± SE for 3 replicate determinations. Click here for file

Additional file 2: Figure S2 Induction of CYP1B1 mRNA by AHR-active pharmaceuticals. MDA-MB-231 cells were treated with DMSO, different concentrations of pharmaceuticals and 10 nM TCDD, and CYP1B1 mRNA levels were determined by real time PCR as outlined in the Methods. Results are expressed as means ± SE for 3 replicate determinations. Click here for file

Additional file 3: Figure S3 Effects of AHR-active pharmaceuticals and TCDD on CYP1A1 and AHR proteins. MDA-MB-231 cells were treated with DMSO, TCDD and AHR-active pharmaceuticals for 24 hr, and whole cell lysates were analyzed by western blots as outlined in the Methods. Click here for file

Additional file 4: Figure S4 Growth inhibition. MDA-MB-231 cells were treated with DMSO, different concentrations of AHR-active pharmaceuticals for 24 hr, and cell growth was determined using MTT assay as outlined in the Methods. Results are expressed as means ± SE for at least 3 replicate determinations. Click here for file
