# Supplementary material for: The aryl hydrocarbon receptor ligand omeprazole inhibits breast cancer cell invasion and metastasis
Source: BMC Cancer. 2014 Jul 9;14:498. doi: 10.1186/1471-2407-14-498 (PMC4226953; doi:10.1186/1471-2407-14-498)
Supplement: Additional file 1: Figure S1 — Induction of CYP1A1 mRNA by AHR-active pharmaceuticals. MDA-MB-231 cells were treated with DMSO, different concentrations of pharmaceuticals and 10 nM TCDD, and CYP1A1 mRNA levels were determined by real time PCR as outlined in the Methods. Results are expressed as means ± SE for 3 replicate determinations. [file 1471-2407-14-498-S1.pdf]

Supplemental Figure 1

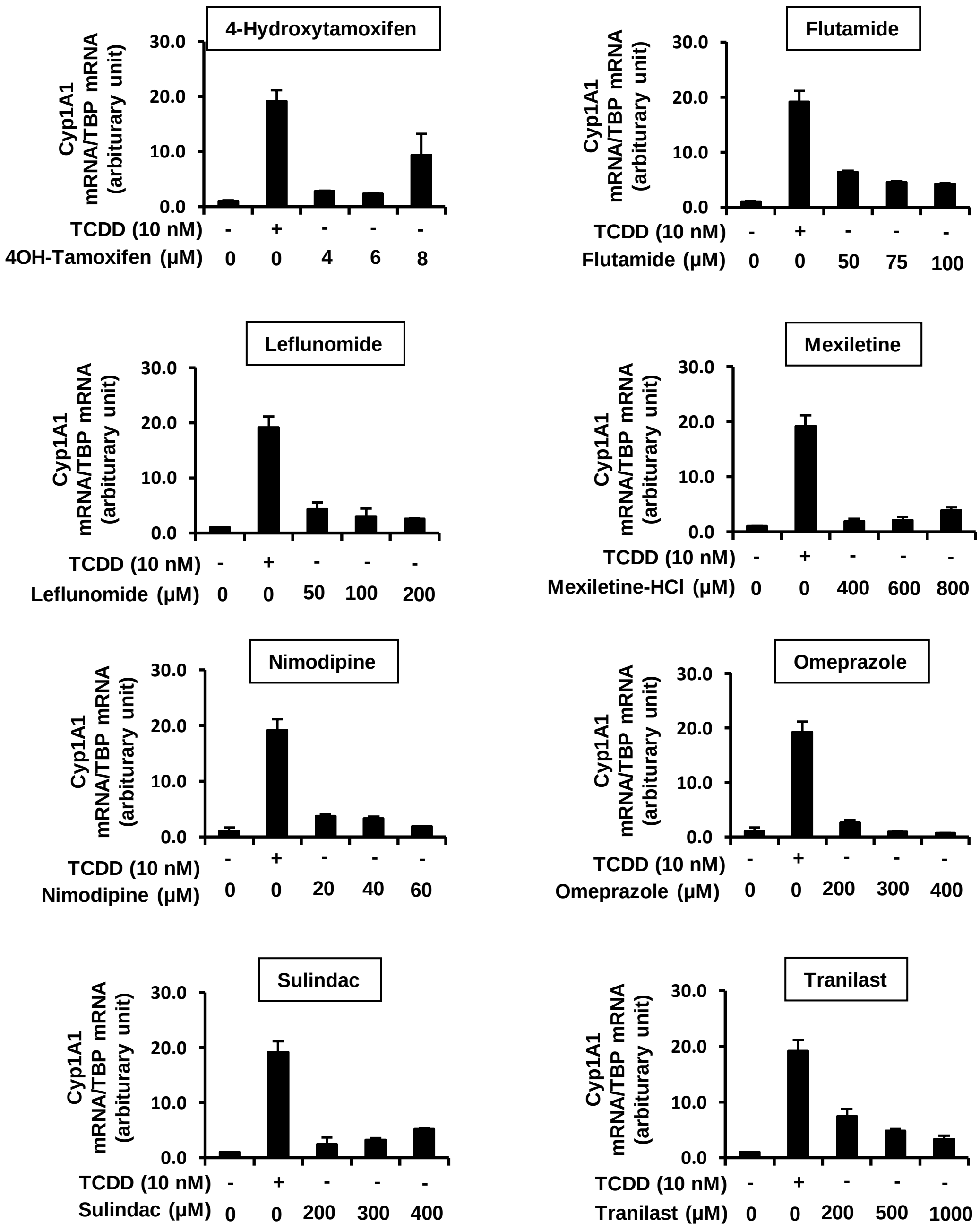

Figure S1. Induction of CYP1A1 mRNA by AHR-active pharmaceuticals. MDA-MB-231 cells were treated with DMSO, different concentrations of pharmaceuticals and 10 nM TCDD, and CYP1A1 mRNA levels were determined by real time PCR as outlined in the Materials and Methods. Results are expressed as means ± SE for 3 replicate determinations.
