# Supplementary material for: The aryl hydrocarbon receptor ligand omeprazole inhibits breast cancer cell invasion and metastasis
Source: BMC Cancer. 2014 Jul 9;14:498. doi: 10.1186/1471-2407-14-498 (PMC4226953; doi:10.1186/1471-2407-14-498)
Supplement: Additional file 3: Figure S3 — Effects of AHR-active pharmaceuticals and TCDD on CYP1A1 and AHR proteins. MDA-MB-231 cells were treated with DMSO, TCDD and AHR-active pharmaceuticals for 24 hr, and whole cell lysates were analyzed by western blots as outlined in the Methods. [file 1471-2407-14-498-S3.pdf]

Supplemental Figure 3

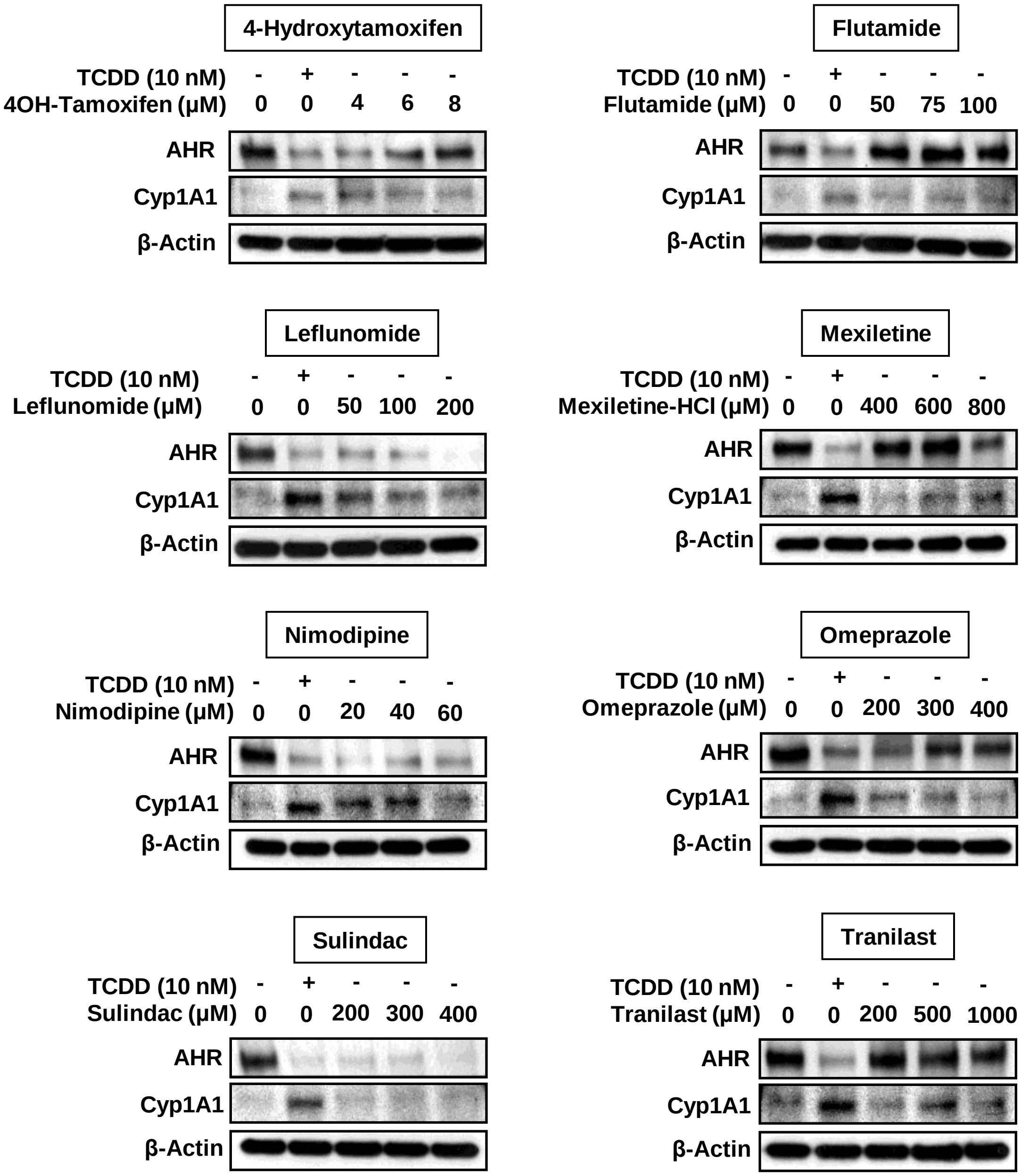

Figure S3. Effects of AHR-active pharmaceuticals and TCDD on CYP1A1 and AHR proteins. MDA-MB-231 cells were treated with DMSO, TCDD and AHR-active pharmaceuticals for 24 hr, and whole cell lysates were analyzed by western blots as outlined in the Materials and Methods.
