# Supplementary material for: The aryl hydrocarbon receptor ligand omeprazole inhibits breast cancer cell invasion and metastasis
Source: BMC Cancer. 2014 Jul 9;14:498. doi: 10.1186/1471-2407-14-498 (PMC4226953; doi:10.1186/1471-2407-14-498)
Supplement: Additional file 4: Figure S4 — Growth inhibition. MDA-MB-231 cells were treated with DMSO, different concentrations of AHR-active pharmaceuticals for 24 hr, and cell growth was determined using MTT assay as outlined in the Methods. Results are expressed as means ± SE for at least 3 replicate determinations. [file 1471-2407-14-498-S4.pdf]

Supplemental Figure 4

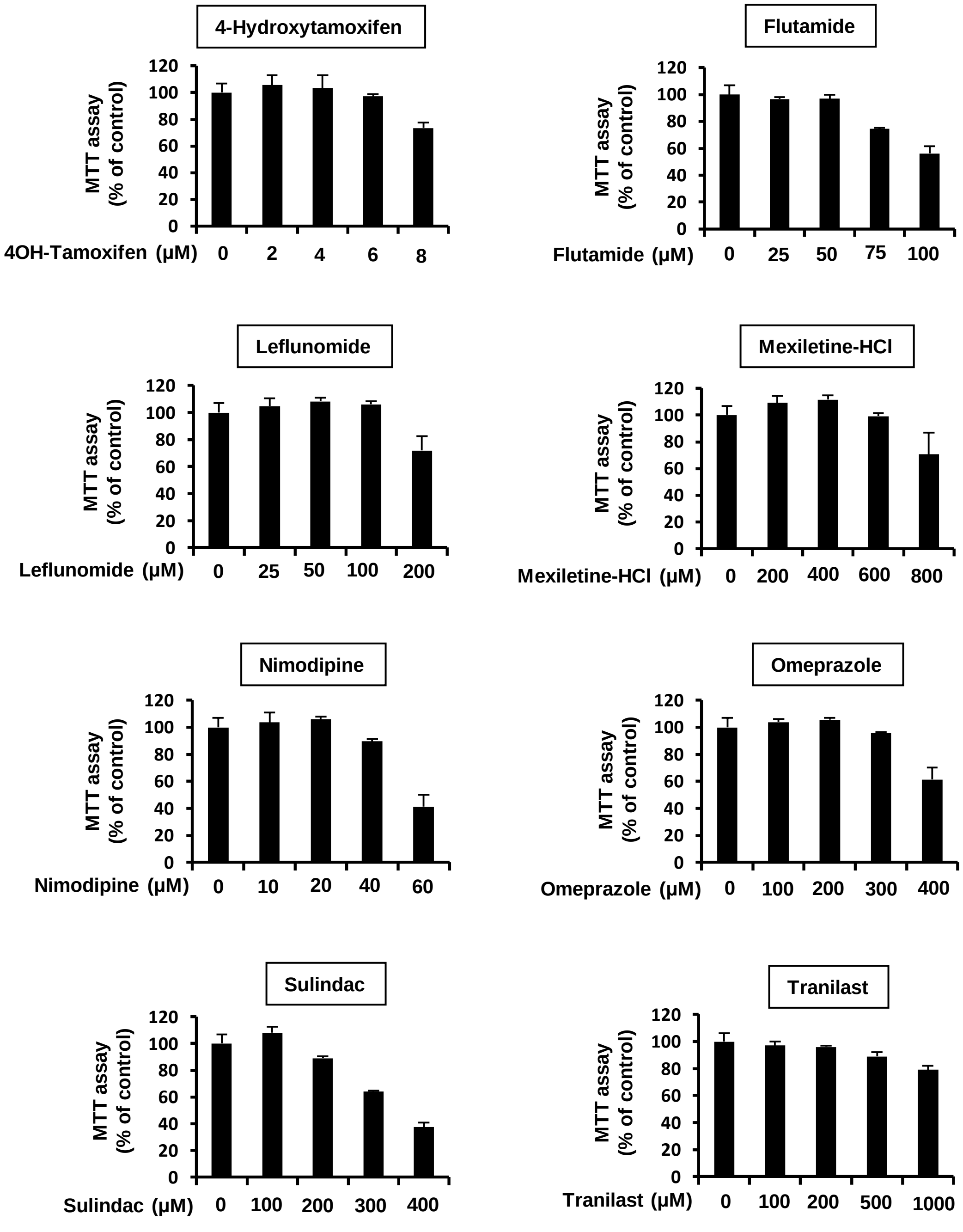

Figure S4. Growth inhibition. MDA-MB-231 cells were treated with DMSO, different concentrations of AHR-active pharmaceuticals for 24 hr, and cell growth was determined using MTT assay as outlined in the Materials and Methods. Results are expressed as means ± SE for at least 3 replicate determinations.
